# Cognitive load associates prolonged P300 latency during target stimulus processing in individuals with mild cognitive impairment

**DOI:** 10.1038/s41598-023-43132-8

**Published:** 2023-09-24

**Authors:** Pinar Demirayak, İlayda Kıyı, Yağmur Özbek İşbitiren, Görsev Yener

**Affiliations:** 1 Civitan International Research Center, University of Alabama at Birmingham, 1719 6th Ave S Suite:252B, Birmingham, AL 35233 USA; 2 Department of Neurobiology, Heersink School of Medicine, University of Alabama at Birmingham, Birmingham, AL USA; 3 Department of Neurosciences, Institute of Health Sciences, Dokuz Eylül University, İzmir, Turkey; 4 Brain Dynamics Multidisciplinary Research Center, Dokuz Eylül University, İzmir, Turkey; 5 Faculty of Medicine, İzmir University of Economics, İzmir, Turkey; 6 İzmir International Biomedicine and Genome Institute, Dokuz Eylül University, Izmir, Turkey

**Keywords:** Alzheimer's disease, Human behaviour

## Abstract

Alterations in P300 amplitude and latency, as well as neuropsychological tests, are informative to detect early signs of the affected high cognitive processing in Mild Cognitive Impairment (MCI). In the present study, we examined P300 latency and amplitude elicited by visual oddball paradigm in 20 participants with MCI and age, education, and sex-matched healthy controls from frontal, central, and parietal midline electrodes. We performed a mixed-design ANOVA to compare P300 amplitude and latency between groups during target and non-target stimulus presentation. We also assessed the correlation between our electrophysiology findings and neuropsychological tests. Our results indicated that in healthy individuals P300 is elicited earlier in target stimulus processing compared to non-target stimulus processing. On the contrary, in the MCI group, P300 latency was increased during target processing compared to non-target stimulus processing. Moreover, P300 latency in target processing is prolonged in the MCI group compared to controls. Also, our correlation results showed a significant correlation between P300 peak latency and amplitude, and attention required cognitive tasks. In conclusion, our results provide evidence that high-order cognitive processes that are involved in stimulus processing slows down in individuals with MCI due to the high working memory demand for neural processing.

## Introduction

Mild cognitive impairment (MCI) is a clinical stage diagnosis used when individuals have symptoms that might be early signs of Alzheimer’s Disease (AD). At this stage, individuals’ with MCI do not display any loss in daily functioning^1,2^. A high percentage of individuals with MCI progress to dementia over a relatively short period of time. Thus, MCI might be considered as a symptomatic pre-dementia stage^3^ that can be divided into four clinical subtypes depending on the presence or absence of episodic memory impairment (amnestic vs non-amnestic) and the number of affected cognitive domains (single-domain vs multiple domains)^4–9^. Individuals with multiple-domain amnestic MCI have impairments not only in memory but also in cognitive abilities including attention, working memory, and executive function impairments^4,5^.Both MCI phenotypes (amnestic vs non-amnestic/single domain vs multiple domain) determine the risk of developing dementia in the future. Multiple-domain and amnestic MCIs have a higher risk of progression from MCI to dementia than single-domain and non-amnestic MCIs, respectively^3,10,11^. Specifically, it is reported that 30% of amnestic MCI cases turn into dementia^12^ and amnestic MCI is considered as a risk factor for Alzheimer's disease (AD), the most common form of dementia. Finding potential MCI biomarkers is crucial to identify this transition period. The identification leads to therapeutic intervention at an early stage of the disease with recently approved treatment options with antiamyloid properties^13–15^. Electroencephalography (EEG) and event-related potential (ERP) recording provide non-invasive methods to identify neural correlates of the disease.

The ERP is a dynamic technique used to understand the cognitive processing of the brain, using waveforms obtained by recording the voltage changes of the working brain over the scalp via EEG during specific neural or psychological processes (such as selective attention, working memory, and semantic processing)^16–18^. The P300 component occurs approximately 300 ms in the positive direction after a specific stimulus, and P300 is observed in attentional tasks when task related stimulus is detected^19^. Some studies showed that MCI patients have longer P300 latency and lower P300 amplitude^20^ when compared to healthy controls^21,22^. In a recent review of visual event potentials by Morrison and colleagues (2019), twelve studies compared P300 amplitude in healthy older adults, in MCI, and/or in AD dementias. In eight studies, they found that P3b amplitude decreased in MCI and AD compared to healthy controls, while four studies did not find any amplitude difference between groups^23^. Another systematic review showed that sixteen of 29 studies indicated no significant amplitude differences between P300 in MCI and healthy controls, while 11 studies showed smaller amplitudes in MCI^24^. Although delay in latency in individuals with AD is frequently shown in the literature, the findings of the P300 latency difference in MCI are inconsistent. Some studies showed no difference in the latency of P300 in MCI compared to healthy controls. However, a few studies suggested a delay in P300 and P3b latency in MCI (For reviews, see^23–25^). These inconsistencies may have been caused by differences in task designs and the heterogeneity of the MCI groups.

The P300 wave has been associated with a range of cognitive processes, including decision-making^26^, memory^27–29^, orienting^30^, and response selection processes when target stimuli need to be discriminated from non-target stimuli^31^. The target selection process is an attention-driven process that involves working memory since participants are required to remember and constantly compare the current stimulus with the target stimulus^32^. Individuals’ high-level cognitive processing capacity is important to determine the allocation of attentional sources to update information or detect a mismatch between expectation and prior knowledge based on the task demands. High cognitive capacity is crucial to resolving the tradeoff to using limited attentional resources appropriately to give potential rapid responses.

It is possible to obtain the P300 with various paradigms, and one of the most widely used is the oddball paradigm. The neural responses related to the detection of the relevant stimulus are commonly studied with oddball paradigms that require the detection of the infrequent stimulus among the trains of frequent stimuli. The P300 component is associated with high cognitive processes that allow individuals to update the information they perceive, making an assumption for the future stimulus and comparing the current stimulus with the task demands to detect targets^32^. Processing the current stimulus, checking if the task demands are met, and mental counting of the target stimulus increase the amount of attentional sources that are allocated to the task. If the cognitive capacity is impaired, it requires greater amounts of attentional resources to be involved and results in reduced P300 amplitude and prolonged P300 latency^33,34^. Since attentional resources that are fundamental for memory processing are limited in individuals with MCI, attention, and memory-required tasks elicit late positive ERP responses due to delayed retrieval^35^. It has been shown that P300 latency changes across the scalp, latency is shorter over frontal areas, whereas it gets longer over parietal areas^36^. Both the availability of the attentional resources and how rapidly subjects can allocate these resources vary with cognitive capacity and behavioral research as well as their relationship with P300 components would give detailed information about the mild cognitive impairment period.

Behavioral studies showed impaired cognitive deficits in working memory, executive functions and attention in individuals with MCI compared to healthy individuals^37–39^. Given the involvement of multiple cognitive domains in neuropsychological assessments, determination of the impaired cognitive deficit is difficult to specify. While semantic processing could be assessed by using a naming test, other contributing cognitive functions such as working memory, attention, visuo-perceptual abilities might also be affected^40^. Some studies indicated that cognitive functions other than memory^41^ such as attention and executive functions^42^ are the first cognitive abilities to be affected. Attention is a core cognitive ability that can modulate activity at various stages of the visual pathways^43–45^. There is growing evidence in the literature showing that this modulation of visual processing is under control of high-order cognitive processing cortical areas^46,47^.

In the present study, we aim to investigate neural correlates of the affected cognitive functions in participants with mild cognitive impairment. Although neural correlates of disruptions in cognitive functioning in MCI have been shown previously, correlations of components of P300 responses during target and non-target visual stimulus processing and neuropsychological tests in multiple-domain amnestic MCI patients have not been investigated. We hypothesized that participants with MCI would have reduced P300 amplitude and prolonged P300 latency in target processing compared to controls. Also, we hypothesized that individuals with MCI would have prolonged P300 latency in target processing than non-target processing possibly due to impaired attentional mechanisms and increased memory load. We further hypothesized that cognitive functions such as executive functioning, working memory and attentional mechanisms are associated with differences in amplitude and latency during target versus non-target stimulus processing.

## Methods

### Participants

Data were acquired from 20 participants (mean age ± sd = 70.65 ± 7.08) with Mild Cognitive Impairment (MCI) according to Petersen’s criteria^1^ recruited from the neurology outpatient clinic of Dokuz Eylul University Medical School Hospital. Twenty age (group comparison (t(38) = 0.169, *p* = 0.867), sex (M/F = 9/11 in both groups) and education (mean education in years ± sd = 10.2 ± 4.99 in MCI, mean education ± sd in HC = 10.3 ± 4.11, group comparison (t(38) = 0.69, *p* = 0.945) matched healthy controls (mean age ± sd = 71 ± 5.97) were recruited from various community resources. The study was approved by the Dokuz Eylul University Non-invasive Research Ethics Committee (#121-GOA) in accordance with the Declaration of Helsinki. Data were collected in accordance with the ethical standards of the Dokuz Eylul University Non-invasive Research Ethics Committee and the Code of Ethics of the World Medical Association (Declaration of Helsinki). All participants provided written informed consent prior to their voluntary participation.

Our inclusion criteria for the MCI group includes subjective memory complaints verified by a relative and accompanied by a memory test score of 1.5 standard deviation less than the mean age norm and a CDR of 0.5 and not fitting the dementia criteria. Individuals who have vascular or trauma related lesions or any neurological or psychiatric disorders were excluded from the study. Also, individuals with depressive comorbidity who score greater than 11 on Geriatric Depression Scale^48^ were excluded from the study. Moreover, participants with regular use of anti-dementia drugs, antidepressants, neuroleptics, anti-epileptic medications, stimulants, opioids, or beta-blockers were excluded from the study. The demographical and neuropsychological characteristics of healthy control and MCI groups were reported in Table 1.

**Table 1:** Demographical and neuropsychological characteristics of healthy control and MCI participants.

	HC (n = 20)	MCI (n = 20)	*p* value
Age (year)^a^	71.00 ± 5.96	70.65 ± 7.08	0.867
Education (year)^a^	10.30 ± 4.11	10.20 ± 4.99	0.945
Gender (F/M)^b^	11/9	11/9	1.000
MMSE^a^	29.31 ± 0.82	25.85 ± 3.30	< 0.001
GDS^a^	6.00 ± 3.71	8.20 ± 5.31	0.144
OVMPT-T^a^	109.25 ± 13.92	68.65 ± 18.75	< 0.001
OVMPT-IR^a^	4.50 ± 1.43	3.65 ± 1.66	0.091
OVMPT-FR^a^	12.65 ± 1.35	5.50 ± 3.62	< 0.001
OVMPT-TR^a^	15.00 ± 0.00	13.35 ± 1.46	< 0.001
DS-Forward^a^	5.10 ± 0.91	4.95 ± 0.97	0.616
DS-Backward^a^	3.90 ± 0.97	3.37 ± 0.68	0.056
Stroop-Int (sec)^a^	47.75 ± 21.43	68.06 ± 38.71	0.052
Animal^a^	22.55 ± 4.03	16.55 ± 2.35	< 0.001
BNT-15^a^	14.92 ± 0.28	13.94 ± 1.70	0.027

Data presented as mean ± standard deviation. ^a^Independent samples t-test; ^b^Chi-square test. *HC* healthy controls, *MCI* mild cognitive impairment, *F* female, *M* male, *MMSE* mini mental state examination, *GDS* geriatric depression scale, *OVMPT-T* oktem verbal memory processes test-total learning, *IR* immediate recall, *FR* free recall, *TR* total recall, *DS* digit span, *Stroop-Int* stroop test interference time, *BNT-15* boston naming test-15 item. *p* < 0.005 considered as significant.

### Experimental paradigm

Participants were asked to sit in a dimly lit, isolated room during the EEG recording. Classical visual oddball paradigm was applied using a simple 10-cd/m^2^ luminance light as the standard and a 40-cd/m^2^ luminance light as the target stimuli. The light appeared at full size on a 19-inch computer monitor with a refresh rate of 60 Hz. The duration of the stimulation was 1000 ms, the probability of the deviant (target) stimuli was 0.33 and, in all paradigms, targets were embedded randomly within a series of standard stimuli (non-target). These stimulation signals were applied randomly, with inter-stimulus intervals varying between 3 and 7 s. A total of 120 stimuli were presented in each data acquisition. In order to assess the neural correlates of cognitive abilities, participants were asked to perform mental counting of the target stimuli and report the total number to the experimenter at the end of the experiment with an allowed error rate of 10%. There is no significant differences between HC (40.55 ± 1.61) and MCI groups (39.75 ± 2.29) [t(38) = 1.279, *p* = 0.209] in accuracy.

### EEG data acquisition and preprocessing

EEG was recorded with 30 Ag/AgCl electrodes mounted in an elastic cap (Easy-cap) according to the international 10–20 system. We used a monopolar montage with two linked earlobe electrodes (A1 + A2) which served as references. The electrooculogram (EOG) from the medial upper- and lateral orbital rims of the right eye was registered to remove artifacts. For the reference electrodes and EOG recordings, Ag/AgCl electrodes were used. All electrode impedances were less than 10 kΩ. The EEG was amplified by means of a Brain Amp 32-channel DC system machine with band limits of 0.01–70 Hz. The EEG was digitized online with a sampling rate of 500 Hz. The recording room was electrically shielded, sound attenuated, and dimly illuminated. A 50 Hz notch filter has been applied offline to eliminate signal electrical interference.

Artifacts were eliminated manually and offline, taking into consideration the EOG recorded from the medial upper and lateral orbital locations of the right eye. The epoch numbers were equalized randomly between the target and non-target visual stimulation conditions.

### Neuropsychological tests

All participants underwent an extensive cognitive and neurological examination. Cognitive tests including The Mini-Mental State Examination (MMSE), Oktem Verbal Memory Processing Test (OVMPT), Semantic Verbal Fluency Test, 15-item Boston Naming Test (BNT), Stroop Test, WMS-R digit span tests were chosen to assess participant’s cognitive abilities. Geriatric Depression Scale (GDS) was performed to exclude individuals who have depression to rule out the effects of depression on behavioral and electrophysiological data.

The general cognitive screening was performed by using the Mini-Mental State Examination^49^. Moreover, short-term and long-term memory were assessed by OVMPT^50^. OVMPT consists of reading a list of 15 words, followed by a free recall after a delay and then recognition for the missing words. To be able to assess semantic fluency we performed the animal naming test^51^. Participants were required to generate words from a given category, in this study namely animals. For language assessment, we used the short form of the Boston Naming Test (15 items). BNT was administered where they were asked to name the presented pictures^52^. In addition, the Stroop test^53^ was administered to measure attention, cognitive flexibility, and executive functions. Participants were asked to read written names of colors which could be in consistent or inconsistent colors. We aim to measure their cognitive flexibility to follow task demands, switch to new rules, and suppress a habitual response. Lastly, the WMS-R digit span test was applied with both forward and backward subtests^54^. Age-related standard scores were derived from MOANS^55^.

### Data analysis

Raw EEG data were first high-pass filtered with a 0.1 Hz Zero phase shift Butterworth filter, and power-line noise was eliminated by a 50 Hz notch filter. Extended Infomax Independent Component Analysis (ICA) was used to remove artifacts of the horizontal and vertical components through saccadic eye movements. Data was further filtered between 0.5 and 30 Hz to obtain ERP waveform. The trials with the target stimuli were segmented into 1000 ms epochs time-locked to stimulus onset including the 200 ms pre-stimulus period. Baseline correction was applied in relation to 200 ms of pre-stimulus. A fully automatic artifact rejection procedure was applied with the following criteria: (a) maximum amplitude in an epoch: ± 70 μV, (b) maximum allowed voltage step: 50 μV/ms, (c) maximum allowed difference in a 200 ms interval: 50 μV, (d) lowest activity in a 100 ms interval: 0.5 μV. The remaining artifact-free epochs were averaged to obtain the P300 waveform. The minimum number of epochs was 20 in the whole sample. Maximum peak amplitude and peak latency were automatically measured between 300 and 600 ms time window from Fz, Cz, and Pz electrode sites. The grand average ERP waveforms of target and non-target stimuli from Fz, Cz, and Pz electrode sites are presented in Results section.

### Statistical analysis

Statistical analyses were performed using SPSS v. 28.0.1.1. Descriptive statistics of frequencies and percentages were used for qualitative variables, and means and standard deviations were used to identify demographics of our sample. Independent samples t-test was used to compare groups for demographic variables (ages, sex, and education) and behavioral assessments (neuropsychological test scores). A 2 × 3 × 2 mixed design ANOVA was performed to investigate the effects of group-level differences on target versus non-target processing from Fz, Cz and Pz electrode sites. The sample was stratified according to condition, electrode distribution, and group. Condition (target vs non-target), and anterior–posterior (AP) distribution (frontal, central, parietal) were included as within-subject factors whereas group (MCI vs HC) was included as between-subject factors in our ANOVA design. Post-hoc tests with Bonferroni correction were applied on ANOVA comparisons of ERP findings. The common approach to simultaneous testing of multiple hypotheses is to construct a multiple comparison procedure (Bonferroni) that controls type I error (family-wise error rate [FWE])^56^. However, it is very conservative procedure thus in larger datasets the greater the chances that actually significant differences will be incorrectly rejected. On the other hand, False Discovery Rate (FDR) approach is expected proportion of the true null hypotheses that are erroneously rejected out of the total number of hypotheses rejected^57^. FDR is more powerful than all previously proposed methods for larger datasets^58^. Thus, for our larger dataset, Pearson's correlation coefficients were computed to assess the linear relationship between electrophysiological and behavioral data. False discovery rate (FDR) procedure was applied to correct for false-positive inflation at multiple comparisons^59^. FDR-corrected *p* values were used as a significance level to test our hypotheses in ERP and neuropsychological test comparisons.

### Ethics approval

These data were collected in accordance with the ethical standards of Dokuz Eylul University.

## Results

We hypothesized that individuals with MCI have prolonged P300 response and reduced P300 amplitude in target processing as it was shown in literature. We found that P300 latency was significantly different between groups, however, P300 amplitude did not differ between the groups. Furthermore, we hypothesized that individuals with MCI would have prolonged P300 latency in target processing than non-target processing possibly due to impaired attentional mechanisms and increased working memory load. As we expected, P300 latency was different between target and non-target processing in the MCI group. We further hypothesized that cognitive functions such as executive functioning, working memory and attentional mechanisms are associated with differences in ERP findings. We found that cognitive functions such as executive functioning, attention and memory are impaired in the MCI group, these cognitive impairments are associated with prolonged P300 latency and reduced P300 amplitude and latency during target versus non-target stimulus processing.

### Peak P300 latency differs between groups during target versus non-target processing

**Figure 1:**
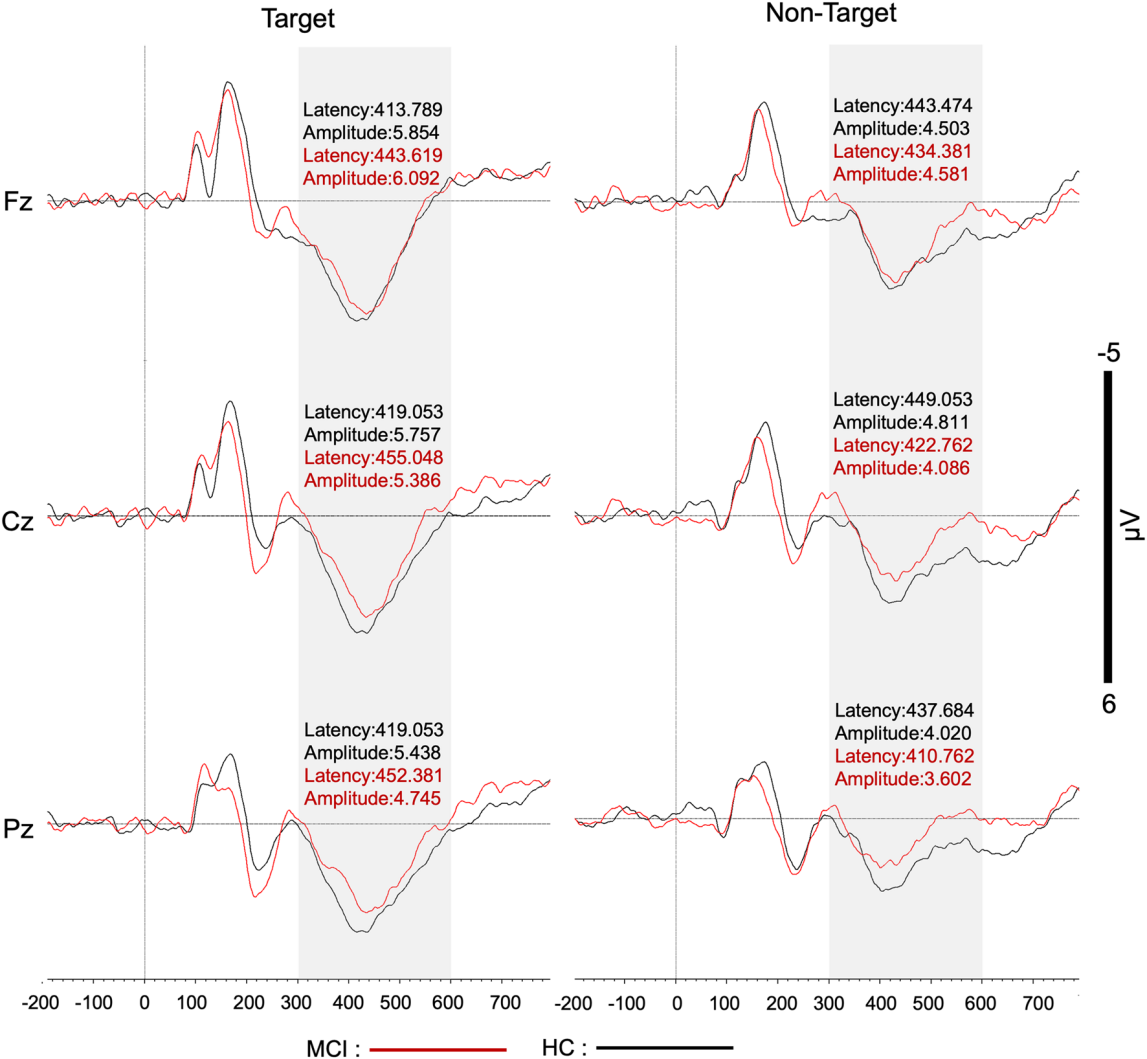
Grand average P300 waveforms from Fz, Cz, and Pz electrodes during target and non-target visual stimulus presentation in both MCI (shown in red) and healthy control (shown in black) groups. Maximum P300 peak amplitude and peak latency were measured between the 300 and 600 ms time window that is shaded in gray after stimulus presentation in our visual oddball paradigm. The mean P300 peak and latency values for each group were added as labels on each graph.

Peak P300 component was averaged during target and non-target visual stimulus presentation after 300–600 ms time window from Fz, Cz and Pz electrodes for both groups. A mixed design 3 × 2 × 2 ANOVA [3 (anterior–posterior electrode localization:Fz, Cz and Pz) × 2 (condition: target vs non-target × 2 (groups: MCI vs HC)] test was performed on peak P300 amplitude and group comparison (F(1,38) = 0.316 *p* = 0.577) did not reach significance level. Nevertheless, when the same test was applied on peak P300 latency, although there was no group difference (F(1,38) = 0.433 *p* = 0.514), condition and group interaction effect was statistically significant (F(1,38) = 8.133 *p* = 0.007**). P300 latency during target versus non-target processing was different between MCI and their matched healthy controls. Post-hoc analyses showed that P300 latency during target and non-target processing is different in the MCI group (*p* = 0.034) and the effect is trending in the healthy control group (*p* = 0.074). Target processing speed in the MCI group is more prolonged than non-target processing. Also, post-hoc analyses showed that P300 latency in target processing between the MCI and their matched healthy control groups are different (*p* = 0.026). No group difference was found in P300 latency in non-target processing (*p* = 0.294). Grand average of P300 responses were shown in Fig. 1 during target and nontarget stimulus presentation for both groups. Average P300 peak amplitude and latency values and their standard deviations were summarized in Table 2.

**Table 2:** Mean and standard deviation of the P300 peak amplitude and latency values from midline electrode channels during target and non-target stimulus presentation in both healthy control and MCI groups.

Stimulus type	Channels	ERP components	HC (n = 20)	MCI (n = 20)
Target	Fz	Amplitude	5.854 ± 2.121	6.092 ± 3.005
		Latency	413.789 ± 29.081	443.619 ± 56.465
	Cz	Amplitude	5.757 ± 1.919	5.386 ± 2.804
		Latency	419.053 ± 30.312	455.048 ± 64.005
	Pz	Amplitude	5.438 ± 1.906	4.745 ± 2.486
		Latency	419.053 ± 43.703	452.381 ± 60.417
NonTarget	Fz	Amplitude	4.503 ± 1.722	4.581 ± 2.339
		Latency	443.474 ± 57.418	434.381 ± 59.979
	Cz	Amplitude	4.811 ± 1.600	4.086 ± 1.882
		Latency	449.053 ± 72.631	422.762 ± 68.210
	Pz	Amplitude	4.020 ± 1.478	3.602 ± 1.776
		Latency	437.684 ± 63.394	410.762 ± 77.801

Data presented as mean ± standard deviation. *HC* healthy controls, *MCI* mild cognitive impairment.

### MCI patients have impairment in multiple cognitive domains

**Figure 2:**
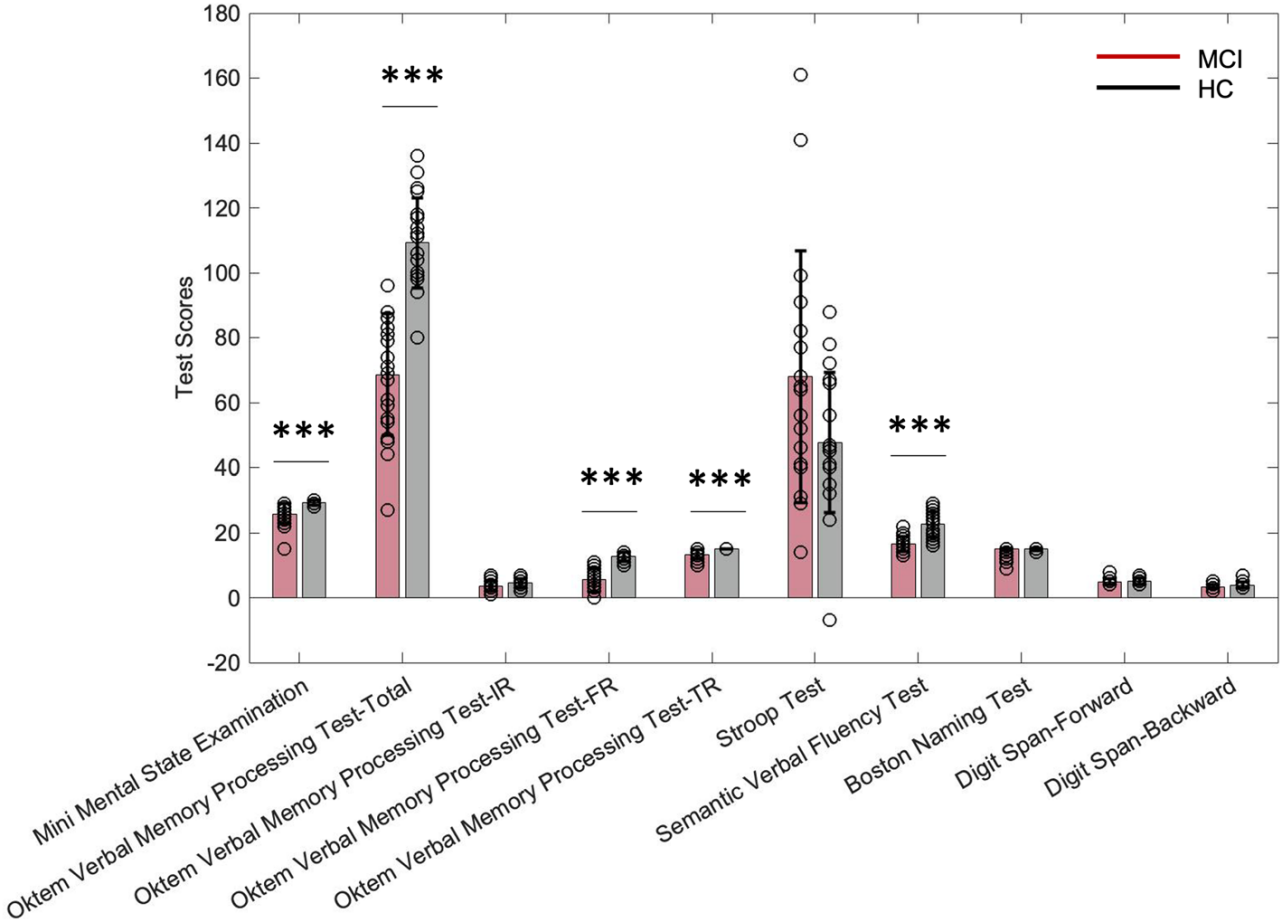
Comparison of cognitive tests between individuals with MCI (shown in red) and their matched healthy controls (shown in black). Comparisons that are significant based on independent sample t test were identified with an asterisk, ***p < 0.001. Bars show mean test scores and error bars represent 士 1 standard error.

MMSE, subtests of the Oktem verbal memory processing test (OVMPT) (immediate recall (IR), free recall (FR), and total recognition (TR)) as well as the total score, Stroop test interference, semantic verbal fluency test, Boston Naming Test and two subtests of Digit Span Test (forward and backward), were assessed in both MCI patient group and control group. The MCI group exhibited significantly worse performance in the MMSE (t(37) = 4.450, *p* < 0.001), OVMPT total score (t(38) = 7.774, *p* < 0.001), free recall (t(38) = 8.277, *p* < 0.001), and total recognition (t(38) = 5.051, *p* < 0.001) subtests of OVMPT and semantic verbal fluency tests than healthy controls (Fig. 2).

### Attention required tasks are negatively correlated with P300 components in MCI group

**Figure 3:**
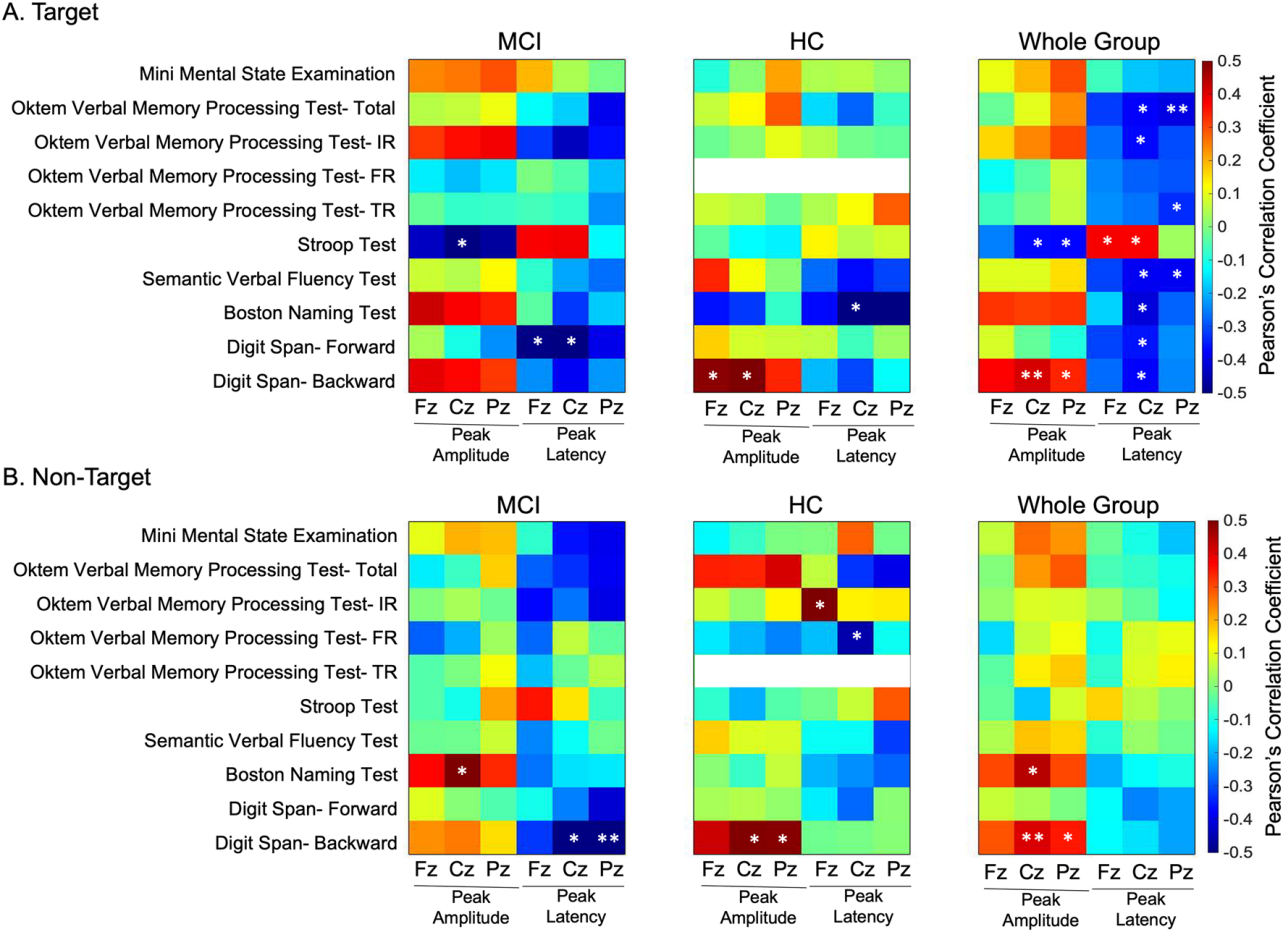
Correlation between P300 peak amplitude, P300 latency, and neuropsychological tests in MCI, healthy control, and whole group during target (**A**) and non-target (**B**) presentation. *p* values less than FDR corrected p criterion are marked(*). All of the healthy controls performed a full score in the verbal memory processing test total recognition, since there is no variation in the score correlation between the test and the P300 component could not be calculated. These results were shown in white color in the figure.

Correlations among cognitive tests and P300 peak amplitude and P300 peak latency during target and non-target processing were summarized in Fig. 3. In target processing, Stroop interference was significantly correlated with P300 peak amplitude from Cz electrode (r = − 0.497, *p* = 0.043*). Also, digit span forward subtest results were negatively correlated with P300 peak latency in Fz (r = − 0.496, *p* = 0.031*) and Cz (r = − 0.51, *p* = 0.026*) electrodes in MCI patients. In the healthy control group, Boston naming test results were negatively correlated with P300 peak latency (r = − 0.601, *p* = 0.03). On the contrary, digit span backward subtest results were positively correlated with P300 peak amplitude in Fz and Cz electrodes in healthy controls (r = 0.485, *p* = 0.03* and r = 0.538, *p* = 0.014* respectively) (Fig. 3A). Correlation between OVMPT total recognition test scores and P300 component could not be assessed in the healthy control group due to the lack of variance. All healthy participants scored the highest score. We also assessed correlation between whole group P300 components and cognitive tests. P300 peak latency was negatively correlated with almost all of the cognitive test scores (P300 peak latency from Cz and OVMPT total score (r = − 0.379, *p* = 0.016*), OVMPT IR (r = − 0.37, *p* = 0.019*), semantic verbal fluency test (r = − 0.389, *p* = 0.013*), Boston naming test (r = − 0.411, *p* = 0.022*), digit span forward test (r = − 0.359, *p* = 0.025) and digit span backward test (r = − 0.378, *p* = 0.018*) in target processing. In addition, OVMPT total score was negatively correlated with P300 peak latency from Pz in whole group comparison (r = − 0.405, *p* = 0.01**) in target processing. Peak P300 latency from Fz (r = 0.382, *p* = 0.02*) and Cz (r = 0.377, *p* = 0.021*) electrodes were positively correlated with Stroop test interference whole group comparison (Fig. 3A). Digit span backward subtest was positively correlated with P300 amplitudes (Fz peak r = 0.412, *p* = 0.009*; Pz peak r = 0.337, *p* = 0.036*). Stroop test scores were negatively correlated with Cz (r = − 0.368, *p* = 0.025*) and Pz (r = − 0.373, *p* = 0.023**) P300 peak amplitudes in target processing (Fig. 3A).

Boston naming test score was positively correlated with Cz P300 peak amplitude (r = 0.515, *p* = 0.029*) in the MCI group during non-target stimulus presentation. Also, digit span backward test is negatively correlated with Cz (r = − 0.525, *p* = 0.021*) and Pz (r = − 0.634, *p* = 0.004**) peak latency (Fig. 3B). In healthy control group, OVMPT IR and Fz peak latency (r = 0.522, *p* = 0.018*), Digit span backward and Cz P300 peak amplitude (r = 0.494, *p* = 0.027*), and Digit span backward and Cz P300 peak amplitude (r = 0.489, *p* = 0.029*) were positively correlated in non-target processing. Furthermore, OVMPT FR and Cz P300 peak latency (r = − 0.458, *p* = 0.042*) were negatively correlated in HC group during non-target stimulus presentation (Fig. 3B). When we assess the correlation between whole group neuropsychological tests and P300 components in non-target processing, Digit span backward subtest is positively correlated with Cz (r = 0.41, *p* = 0.01**) and Pz (r = 0.349, *p* = 0.029*) peak amplitude. Moreover, Boston Naming Test score is positively correlated with Cz (r = 0.443, *p* = 0.013*) P300 peak amplitude (Fig. 3B).

## Discussion

In this study, we investigated behavioral and neural correlates of cognitive functions in individuals with mild cognitive impairment (MCI). In agreement with the literature, participants with MCI showed decreased performance in the MMSE, verbal memory processing, and semantic fluency tests. Furthermore, our results indicated that the P300 wave is elicited earlier during target visual stimulus processing than non-target stimulus in healthy participants. Nevertheless, the P300 wave is elicited later in target processing than non-target processing in participants with MCI. We investigated how impaired cognitive abilities, namely verbal working memory, verbal memory, semantic memory, executive functions, and attentional mechanisms are related to the amplitude and latency values of P300 elicited from midline electrodes. We found that the executive functioning required task (Stroop test) is negatively correlated with P300 peak amplitudes in the MCI group during target processing. In addition, the attention task (Digit Span Test) is negatively correlated with P300 latency in the MCI group in both target and non-target processing. Moreover, P300 peak latency and Boston Naming Test that assess verbal skills and semantic memory are positively correlated in the MCI group.

Although other studies showed individuals with MCI have prolonged P300 latencies and smaller P300 amplitudes during visual tasks, our results showed only prolonged P300 latency during target processing in the MCI group compared to their matched controls. It has been previously reported that even healthy aging is associated with decreased P300 amplitude and prolonged P300 latency during a visual task^60–63^. However, previous work from our lab indicated that P300 amplitudes are affected by age but not the P300 latency during visual oddball paradigm^64^. In addition to the aging effect on P300 latency, neural correlates of MCI slow down the P300 response even more in the MCI group. What does P300 latency indicate? P300 latency is one of the most common aspects of the P300 wave that is thought to reflect post-stimulus information processing^32,63,65,66^ including classification speed^32^ and executive functions (attention, memory)^67,68^. Prolonged P300 latency in target processing compared to non-target processing in the MCI group and prolonged P300 response in target processing in the MCI group compared to controls indicate slower processing in the MCI group when they need to identify and classify the target stimulus. Thus, our results provide evidence that high-order cognitive processes (i.e. executive functioning and memory) that are involved in stimulus processing slow down in individuals with MCI due to the high working memory demand for neural processing.

Executive function refers to orchestrating and guiding behavior in accordance with internal goals and external demands. Attention has a key role in executive functions to guide action and maintain or suppress information^69^. P300 amplitude is related to the allocation of attentional resources during cognitively demanding tasks^70,71^ and deficits in attentional control have been shown in MCI in the literature^72^. Although Cespon et al. (2014) investigated executive processes in both single-domain and multiple-domain MCIs by using the Simon task and did not find a difference in P300 components between groups^2^, Zurron et al. (2018) tested this phenomenon by using three different executive control tasks, namely Stroop, Simon, and Go/NoGo^19^. They found a prolonged P300 response, particularly in multiple-domain amnestic MCI patients, in all tasks, and the allocation of neural resources for attention was weaker in amnestic MCIs. Similarly, our patients showed cognitive deficits in multiple domains, including memory compared to controls, and we found slower processing speed during target stimulus presentation. Our results indicate impairments in attentional processing in MCI patients. In addition, anterior cingulate cortex and dorsolateral prefrontal cortex areas are related to attention and are activated during top-down (feedback regulatory activity from high-order brain areas) attentional control^73^. These suggest that attentional control is engaged in the visual oddball paradigm and in cognitive tests. Increased P300 latency and its correlation with the Digit Span task also indicate the impairment in attentional control in the MCI group in our study.

Recent neuroimaging studies have shown that the primary visual cortex activity is not limited to retinal sensory input processing, it can also be driven by changes in attention^47,74–76^, and can reflect feedback signals from high-order areas^77,78^. Fronto-parietal network (FPN) is important for directing attentional control^79^. Healthy functional brain networks, such as FPN, show flexibility in neural responses as the brain responds adaptively from moment to moment depending on current goals (flexibility), rather than remaining inflexible. Studies on functional networks showed that individuals with MCI have functional disconnection within the FPN compared to healthy controls^80,81^. Allocation of attentional resources is required to update the perceived information, compare the perceived stimulus with the task demands, and discriminate targets from non-targets. Harper et al. (2017) showed that fronto-parietal network connectivity is related to attention, action selection and target-related memory updating during visual oddball task^82^. Our results indicated that P300 response is delayed in target processing in individuals with MCI than healthy controls. Also, we found that P300 response elicited later in target processing than non-target processing. Disruptions in FPN functional connections may lead to impaired attentional control in individuals with MCI resulting in slower neural processing of target stimuli. Similarly negative correlations between digit span subtasks and P300 latency, and Stroop interference and P300 amplitude indicate impairment in attentional mechanisms in the MCI group. Although they measure more than one cognitive ability, attentional control is a core mechanism to perform tasks. In addition, other cognitive deficits that are experienced by individuals, such as attentional control, may mistakenly be attributed to “poor memory”^83^. Thus, the attentional control mechanism is difficult to untangle in individuals who have memory impairment.

We found that Oktem verbal memory processing test free recall and total recognition subscores and semantic verbal fluency scores were lower in the MCI group compared to their matched healthy control group. Verbal memory processing is related to executive functioning but not related to episodic and semantic memory functioning in healthy aging individuals^84,85^. Similarly, as discussed previously our electrophysiological results and their correlations with cognitive tests indicated impaired executive functioning, especially attentional control in MCI group. Oktem memory processing test free recall and total recognition subscores allow us to assess long term memory as well as attention^50^. Having reduced scores in delayed recall and recognition scores despite similar immediate recall subscores in MCI group compared to control group indicates preserved short term memory and impaired long term memory retrieval in the patient group.

One of the early signs of the MCI is the appearance of neurofibrillary tangles in anterior parahippocampal regions, including ectorhinal, perirhinal and entorhinal areas of the medial temporal lobes^86^. Animal studies showed that these areas are related to recognition memory and executive function^87^. Moreover, electrophysiological studies showed that the inferior temporal^88^ and hippocampus^89^ are P300 generator areas during target detection in visual stimulation. Thus, P300 response and impairment in attentional mechanisms might be affected by neuropathology of the AD in individuals with MCI. Possible neurofibrillary formation in these areas might be a leading factor for our results.

Our study investigated the association between cognitive tests and electrophysiological responses during the visual oddball task. Both behavioral outcomes and neural findings indicated that cognitive processes such as attentional maintenance, executive functioning as well as memory deficits were affected in participants with MCI. Target detection processing increases the cognitive load in participants and thus underlying impaired cognitive abilities slow down neural processing in the MCI group.

## Conclusion

Mild cognitive impairment (MCI) is a transitional state between healthy cognitive aging and Alzheimer’s Disease. Several studies have documented an increased rate of progression to dementia and Alzheimer’s Disease in individuals with MCI. Thus, identification of this transitional state is critical for possible interventions. By using cognitive assessment tools and event-related potentials, particularly P300, we assessed the relationship between cognitive abilities and their neural correlates in the present study. Our study showed that while healthy individuals have earlier P300 responses in target stimulus processing compared to non-target processing, individuals with MCI have prolonged P300 responses in target stimulus processing compared to non-target processing. Also, negative correlations between P300 peak latency and Digit Span subtests; and P300 peak amplitude and Stroop test interference were found in the MCI group. Altogether, our results revealed that individuals with MCI have reduced target processing speed when working memory load is high, compared to their matched healthy controls. Behavioral correlates of these neural response findings support impaired executive functioning, memory, and attentional control abilities in participants with MCI.

## Data Availability

All code used in these analyses are available at https://github.com/pdemirayak/Demirayak_2023_P300.
